# A global framework for action to improve the primary care response to chronic non-communicable diseases: a solution to a neglected problem

**DOI:** 10.1186/1471-2458-9-355

**Published:** 2009-09-22

**Authors:** Dermot Maher, Anthony D Harries, Rony Zachariah, Don Enarson

**Affiliations:** 1 Medical Research Council/Uganda Virus Research Institute (MRC/UVRI) Uganda Research Unit on AIDS, PO Box 49 Entebbe, Uganda; 2 International Union Against Tuberculosis and Lung Disease, Old Inn Cottage, Vears Lane, Colden Common, Winchester SO21 1TQ, UK; 3 Medecins Sans Frontieres, Medical Department, Brussels Operational Centre, Brussels, Belgium; 4 International Union Against Tuberculosis and Lung Disease, Paris, France

## Abstract

**Background:**

Although in developing countries the burden of morbidity and mortality due to infectious diseases has often overshadowed that due to chronic non-communicable diseases (NCDs), there is evidence now of a shift of attention to NCDs.

**Discussion:**

Decreasing the chronic NCD burden requires a two-pronged approach: implementation of the multisectoral policies aimed at decreasing population-level risks for NCDs, and effective and affordable delivery of primary care interventions for patients with chronic NCDs. The primary care response to common NCDs is often unstructured and inadequate. We therefore propose a programmatic, standardized approach to the delivery of primary care interventions for patients with NCDs, with a focus on hypertension, diabetes mellitus, chronic airflow obstruction, and obesity. The benefits of this approach will extend to patients with related conditions, e.g. those with chronic kidney disease caused by hypertension or diabetes. This framework for a "public health approach" is informed by experience of scaling up interventions for chronic infectious diseases (tuberculosis and HIV). The lessons learned from progress in rolling out these interventions include the importance of gaining political commitment, developing a robust strategy, delivering standardised interventions, and ensuring rigorous monitoring and evaluation of progress towards defined targets.

The goal of the framework is to reduce the burden of morbidity, disability and premature mortality related to NCDs through a primary care strategy which has three elements: 1) identify and address modifiable risk factors, 2) screen for common NCDs and 3) and diagnose, treat and follow-up patients with common NCDs using standard protocols. The proposed framework for NCDs borrows the same elements as those developed for tuberculosis control, comprising a goal, strategy and targets for NCD control, a package of interventions for quality care, key operations for national implementation of these interventions (political commitment, case-finding among people attending primary care services, standardised diagnostic and treatment protocols, regular drug supply, and systematic monitoring and evaluation), and indicators to measure progress towards increasing the impact of primary care interventions on chronic NCDs. The framework needs evaluation, then adaptation in different settings.

**Summary:**

A framework for a programmatic "public health approach" has the potential to improve on the current unstructured approach to primary care of people with chronic NCDs. Research to establish the cost, value and feasibility of implementing the framework will pave the way for international support to extend the benefit of this approach to the millions of people worldwide with chronic NCDs.

## Background

The international health focus in the past two decades has been on the problem of infectious diseases, with non-communicable diseases (NCDs) given a low priority at global level [1]. However recognition is increasing of the double burden in developing countries of chronic communicable diseases (e.g. tuberculosis and HIV) and chronic NCDs [2], based on emerging country-level evidence [3]. Attention to chronic NCDs is now increasing for several reasons: 1) they have a huge negative economic impact [4] and represent a significant impediment to human development [5]; 2) the effects of globalisation are likely to have a particular impact on chronic NCDs, including diabetes, hypertension, smoking-related conditions and obesity; and 3) recent progress in mobilising funds and improving the response to infectious diseases (especially HIV/AIDS, tuberculosis and malaria) has enabled a shift to a broader global health outlook.

The main international focus is on those chronic NCDs (diabetes, hypertension, chronic airflow obstruction and obesity) which share the following characteristics: 1) they are becoming more common as a consequence of the effects of globalisation; 2) enough preliminary data on these conditions are available to justify the conclusion that they are contributing to epidemiological transition (a double burden of communicable diseases and non-communicable diseases) in a wide range of low- and middle-income countries; 3) they share common risk factors which are potentially amenable to behavioural modification (tobacco use, unhealthy diets, physical inactivity, and harmful use of alcohol) [6]; 4) they can be detected using simple tests available (or potentially readily available) in primary care settings in low-income countries: hypertension (sphygmomanometer), chronic airflow obstruction (peak expiratory flow meter), diabetes (urine or blood glucose) and obesity (weight and height); 5) they can be managed in typical primary care settings in middle- and low-income countries; 6) the benefits of prevention and care extend to related conditions of public health importance, e.g. chronic kidney disease (often caused by hypertension or diabetes); and 7) they are the focus of World Health Organization efforts, such as the launch of the new global initiative in July 2009, to try to ensure that NCDs are urgently accorded greater priority in the health and development policies of poor nations and on global aid agendas [8]. If the framework for the selected chronic NCDs proves useful, it may pave the way for structured approaches to the prevention and management of other chronic NCDs such as chronic kidney disease, chronic liver disease and chronic organic brain syndromes (e.g. the dementias).

Recent estimates and projections have revealed the extent of the global burden of chronic NCDs. The World Health Organization (WHO) predicts a 17% increase in global NCD deaths over the next decade, with the greatest increase in Africa (27%) [8]. In 2007, it was estimated that there were 246 million people living with diabetes mellitus, 6 million new cases and 3.8 million deaths, with 70% of these patients living in the developing world [9]. In 2000, there were an estimated 972 million people with hypertension, 65% of whom lived in the developing world, with the number predicted to grow to 1.5 billion by 2025 [10]. Chronic obstructive pulmonary disease similarly affects large numbers of people with an estimated 300 million living with asthma [11] and 61 million living with chronic airflow obstruction [12], with three-quarters of the patients living in Asia and Africa. The chronic NCD burden is likely to increase further as scaled-up programmes of antiretroviral treatment (ART) of HIV-infected people lead to reduced HIV/AIDS mortality and to possible metabolic side-effects resulting from life-long ART [13].

A worldwide goal for the prevention and control of NCDs has been proposed to complement the Millennium Development Goals, with the accompanying target of an additional 2% per year reduction in death rates attributable to the main chronic diseases (heart disease, stroke, cancer, diabetes, and chronic respiratory diseases) [14]. WHO has developed a global strategy and action plan for the prevention and control of NCDs [8]. This comprehensive multisectoral approach covers the development of national policy frameworks, establishment of programmes, building of capacity for an effective national response, monitoring and evaluation at different levels, and promotion of research, but does not include a framework for action to improve the primary care response to chronic NCDs. Although primary care is the health service entry point for the vast majority of people with NCDs and therefore plays a key role in the delivery of prevention and care interventions [15], the primary care response to common NCDs is often unstructured and inadequate. There is an urgent need for an effective and affordable framework for use and adaptation by countries to improve the delivery of interventions for patients with chronic NCDs by primary care providers, including those in the government services (whether Ministry of Health services or not, e.g., social-security schemes, prisons, military) and non-government services (e.g., non-governmental organisations, and private practitioners). In practice in many developing countries, secondary and tertiary care institutions often also provide primary care.

Chronic NCDs in developing countries have often been neglected despite their huge burden of morbidity and mortality. Efforts to decrease population-level risks for NCDs need to be accompanied by action to improve the delivery of primary care interventions for people with NCDs. We propose a framework for action to ensure an effective and affordable primary care response to chronic NCDs.

## Discussion

### Rationale for a global framework for action to improve the primary care response to chronic NCDs

Health care delivery systems in developing countries are generally less well oriented towards dealing with chronic NCDs than with infectious diseases. The approach is often unstructured, lacks systematic follow-up and monitoring of chronic clinical care, and provides little information about morbidity or mortality. Moreover, access to essential supplies is often limited and comes at a relatively high cost [16]. This situation is common in developing countries in sub-Saharan Africa [17] and other regions. A lack of quality health information is one of the main obstacles in developing national strategies for an effective response to NCDs [18]. There is a need to document the baseline and future trends in burden of NCDs which may be exacerbated by the effects of globalisation, including global climate change. This information on NCDs is needed for purposes of planning local, national and international responses to changing patterns of disease burden, and monitoring the impact of these responses on disease burden.

Suggested ways to improve the primary health care response to chronic NCDs include: a) the integrated management of chronic NCDs with that of chronic communicable diseases [19], e.g. as demonstrated in Cambodia with HIV, diabetes and hypertension [20]; b) the development of chronic care services that cut across conventional categories of infectious diseases and NCDs [21]; and c) the incorporation of indicators of programme performance and access to services [22]. The standard approach to diagnosis and treatment, and evaluation and reporting of treatment outcomes, which has been a cornerstone of the international strategy to control tuberculosis [23], can be adapted for NCDs - simple systems can be established for monitoring and evaluation of outcomes of patients and of programme performance (through quarterly and cumulative cohort analyses of the numbers of cases registered and their outcomes), as has been shown with the reporting of ART scale-up in Malawi [24]. The importance of this approach to monitoring and evaluation is reflected by Objective 6 of the WHO Action Plan "to monitor non-communicable diseases and their determinants and evaluate progress..." [8]. The establishment of multidisciplinary chronic disease clinics using standardised approaches to patient management could bring efficiency gains, especially in providing continuity of care [25] (an important element of quality care), long-term adherence support [26] and social support, and may help to decrease the stigma often associated with HIV and tuberculosis.

Improvements on the current situation are likely with a programmatic, standardized approach to the delivery of primary care interventions for patients with NCDs, incorporating the above suggestions. The framework for this "public health approach" is designed to improve the management of the vast majority of people with NCDs who can be managed satisfactorily in primary care. The framework also incorporates referral of people with NCDs needing more complex management in secondary or tertiary care. The proposed framework is informed by experience of scaling up interventions for chronic infectious diseases [24]. The lessons learned from progress in rolling out interventions for the diagnosis and treatment of tuberculosis [27] and HIV infection [28] include the importance of gaining political commitment, developing a robust strategy, delivering standardised interventions, ensuring rigorous monitoring and evaluation of progress towards defined targets, and setting ambitious targets which serve as an aspiration for rallying stakeholders in their efforts and helping to increase resources for the global response. The proposed framework for NCDs borrows the same elements as those developed for tuberculosis control [29], comprising a goal, strategy and targets for NCD control, a package of interventions for quality care, key operations for national implementation of these interventions, and indicators to measure progress towards increasing the impact of primary care interventions on chronic NCDs. The generic global framework can be adapted at national level and validated for particular settings, with operational evaluation of feasibility, effectiveness, cost and cost-effectiveness.

### A proposed global framework for the primary care response to chronic NCDs

#### Goal

To reduce the burden of morbidity, disability and premature mortality related to NCDs.

#### Strategy for the primary care contribution to NCD control

Among all those seen in primary care, 1) identify and address modifiable risk factors, 2) screen for common NCDs and 3) diagnose, treat, follow-up and when necessary refer patients with common NCDs using standard protocols.

In many countries, a substantial proportion of the population accesses the primary care system each year. Primary care is an important site for chronic disease prevention as well as care, and those accessing primary care represent a target group for delivery of prevention and care interventions. To complement the multisectoral measures aimed at primary prevention of NCDs, everyone seen in primary care should be assessed for common risk factors such as smoking, alcohol and obesity, and counseled on lifestyle modification. Low-cost, rapid and reliable means of screening are available for common NCDs, e.g. hypertension (automatic sphygmomanometer), chronic airflow obstruction (peak expiratory flow meter), diabetes (urine or finger-prick dipsticks) and obesity (weighing scales and height measuring rod to enable calculation of body mass index). A structured approach to delivering quality care for patients with common NCDs involves the use of simple standard protocols for diagnosis, treatment, follow-up and referral.

#### Targets

1. To assess 90% of patients presenting to primary health care facilities for three common modifiable risk factors for NCD - namely, cigarette smoking, alcohol and obesity - and to address those risk factors when present.

2. To screen 90% of all patients presenting to primary health care facilities at least once each year for common NCDs using simple tests for hypertension (automatic sphygmomanometer), chronic airflow obstruction (peak expiratory flow meter), diabetes (urine or blood glucose, with proteinuria also detectable by dipstick testing of the urine) and obesity (weight and height).

3. To ensure that 90% of patients diagnosed with hypertension, diabetes, obesity, and chronic airflow obstruction are managed and continue within life-long structured and monitored primary care.

#### Package of interventions for quality care

##### 1. Political commitment

Chronic diseases require a (chronic) sustained response and political commitment. Sustained government commitment to comprehensive health system strengthening should include commitment to addressing the problem of NCDs. The government has several key roles, including leadership, stewardship, resource mobilisation and allocation, policy development and implementation, and health workforce development.

##### 2. Case-finding among people attending primary care services

This is a central part of the strategy for the primary care contribution to NCD control. Those accessing primary care represent a target group for delivery of prevention and care interventions. The linked delivery of prevention and care interventions is important: providing care without prevention and vice versa represent missed opportunities.

##### 3. Standardised diagnostic and treatment protocols

The use of simple standard protocols for diagnosis, treatment, follow-up and, when necessary, referral of patients with common NCDs can ensure a structured approach to delivering quality care. Generic protocols for standard case management, using simple and inexpensive tools and drugs, should be adapted at national level and validated for the particular circumstances in particular settings.

##### 4. Regular drug supply

A regular supply of essential drugs is necessary for the quality care of people with NCDs, since an individual patient's clinical course will deteriorate if treatment is interrupted.

##### 5. Systematic monitoring and evaluation

Quarterly and cumulative cohort analyses of the numbers of cases registered and their outcomes are the vital signs of NCD management. Key indicators are: the numbers of patients registered and retained on treatment, and the primary outcomes of alive, dead, stopped treatment, lost to follow-up and transferred to another facility. The number of new cases started on treatment in any given time period represents the new incident cases, and provides a gauge of access to treatment and case detection if the number of expected cases is known or estimated. The cumulative number of patients retained alive and on treatment represents the burden of disease, the data being essential for proper planning of logistics and accurate drug forecasting. Cumulative numbers who have died, have been lost to follow-up or who have stopped treatment provide a measure of programme performance.

### Key operations for national implementation of these interventions

#### 1. Political commitment

1.1 Establish an adequately staffed and funded NCDs and health promotion unit within the Ministry of Health, with guidance on evidence-based policy from a National Advisory Committee. Health promotion includes community sensitisation on NCDs.

1.2 Mobilise and coordinate the investment of funds in strengthening the primary care system to enable delivery of quality care for people with NCDs. Areas of investment include human resources, health care infrastructure, secure supply of materials for diagnosis and of drugs, and record-keeping.

1.3 Develop and disseminate a manual for the strengthened primary care response to NCDs, to be used as a policy reference guide and training tool.

#### 2. Case-finding among people attending primary care services

2.1 Train primary care staff to be able to deliver the strategy for the primary care response to NCDs. Staff should take the opportunity when people are seen in primary care to: 1) identify and address modifiable risk factors, 2) screen for common NCDs, and 3) diagnose, treat and follow-up patients with common NCDs using standard protocols.

2.2 Ensure the availability of the essential materials for diagnosis, treatment and record-keeping which primary care staff need to deliver the strategy.

#### 3. Standardised diagnostic and treatment protocols

3.1 Develop or apply established simple standard protocols for diagnosis, treatment, follow-up and, when necessary, referral of patients with common NCDs which can ensure a structured approach to delivering quality care.

3.2 Ensure the protocols are validated to reflect the approach which is relevant to those NCDs which are common in a particular country, and updated to reflect changing circumstances.

#### 4. Regular drug supply

4.1 Strengthen the national system of drug procurement and delivery to ensure regular and reliable delivery of essential drugs to primary care facilities.

4.2 Base the forecasting of drug requirements on the systematic registration of patients and recording and reporting of their outcomes.

4.3 Obtain access to quality-assured, affordable drugs through manufacture of appropriate generic formulations (suitable for storage under local conditions) and pooled procurement mechanisms. The success of the drug supply system established along these lines through the Global Drug Facility for anti-tuberculosis drugs [30] is now being extended to the supply of drugs for chronic respiratory diseases through the Asthma Drug Facility and may be further extended to drugs for other NCDs.

#### 5. Systematic monitoring and evaluation

5.1 Ensure an efficient system for data collection, entry and management, which enables assessment of clinical progress of individual patients and a comprehensive assessment of the burden of chronic NCDs and its response to health system interventions.

5.2 Design and implement a patient-held record (e.g. "health passport") which indicates the essential information for chronic care - the key clinical findings, main diagnoses, treatments, and appointments for review (including referral) - to complement the recommended record-keeping system for health facilities (as a fail-safe in case the health facility system doesn't work and as an aide-memoire for the patient).

5.3 Design and implement a user-friendly and reliable record-keeping system for health facilities, which may be entirely paper-based, paper-based with a computer-based system for data aggregation and cohort reporting, or entirely computer-based. Introduction of robust, easy-to-use, touch screen computer systems in primary care facilities could enable rapid and efficient record-keeping by staff, and ready access to data for analysis.

5.4 Use strategic information (obtained from routine recording and reporting) to guide improvement in the quality of patient care at the point of care delivery (as is the case, for example, for the recommended system of routine recording and reporting of patients' response to anti-tuberculosis treatment) [31].

Indicators to measure progress towards increased impact of primary care interventions on chronic NCDs

Proposed indicators that could be used at country level to measure progress in NCD management include:

1) a national NCD policy framework that is available in the Ministry of Health;

2) the number of administrative areas in the country that regularly report on the incidence and prevalence of NCD cases reported in primary care;

3) the number of new incident cases reported in primary care over defined time periods;

4) the number of patients alive and on standardised nationally approved therapy (i.e. prevalence of those receiving care) at defined moments in time.

### Next steps

The proposed framework needs to be evaluated and implementation priorities determined in different settings according to the prevailing NCD epidemiology. A programme of health system research is urgently needed to establish the feasibility of implementation, and its cost, cost-effectiveness and acceptability [32]. Such research is responsive to the needs of developing countries [33], and is in line with that proposed by the representatives of health policy and research agencies who developed the "Grand challenges in chronic non-communicable diseases" [34]. Research on local implementation, e.g. at district level, is necessary to support effective national scale-up [35]. The approach in a particular setting depends on assessment of the local health structure, e.g. regarding the role of district hospitals in supporting local primary care facilities, and of human resources. A research approach of "learning by doing" will help to address the many practical issues concerning the required human and infrastructure resources, especially in handling increased NCD case-finding, and the acceptability and logistics of providing integrated care, e.g. clinics for chronic NCDs and chronic infectious diseases. In addition to the information generated by the proposed framework on NCDs among people accessing primary care, NCD epidemiological surveillance is needed to establish the population-level burden of NCDs and assess the proportion of cases detected and managed by the health system.

The Director-General of WHO has recently highlighted the importance of strengthened health systems based on primary care as "the route to greater efficiency and fairness in health care and greater security in the health sector and beyond" [36]. This vision of primary care must encompass improved delivery of interventions for the many people with NCDs seen in primary care. Providing affordable and effective primary care to the often large and increasing numbers of people with NCDs in developing countries will be an immense challenge. The scale of this challenge should be taken not as discouragement, but as a reason for action, in mobilising the wide range of stakeholders involved, finding the resources, and taking the necessary steps to meet the challenge. Implementation of the framework should capitalise on synergies with other disease control efforts in addressing the necessary human and infrastructure resources required to provide quality care, as part of strengthening health systems. Set against the cost of action, the cost of inaction on NCDs is measured in terms of the human toll of illness, disability and death, and the economic toll of lost productivity and development potential.

### Summary

• In many developing countries the current approach to delivery of primary care interventions for people with chronic NCDs is often unstructured and inadequate.

• Informed by lessons learned from progress in scaling up interventions for tuberculosis and HIV, a framework for a "public health approach" has the potential to provide an effective and affordable solution to this neglected problem.

• The elements of the proposed framework for NCDs comprise a goal, strategy and targets, a package of interventions for quality care, key operations for national implementation of these interventions, and indicators to measure progress towards increasing the impact of primary care interventions on chronic NCDs.

• If implementation of the proposed framework is found to be feasible, effective and cost-effective, international support can be mobilised to extend the benefit of this approach to the millions of people worldwide with chronic NCDs.

## Competing interests

The authors declare that they have no competing interests.

## Authors' contributions

DM had the idea for the paper and drafted the manuscript. AH, RZ and DE helped to develop the idea and to draft the manuscript. All authors read and approved the final manuscript.

## Pre-publication history

The pre-publication history for this paper can be accessed here:


